# Bioactive Glass-Based Materials for Soft and Hard Tissue Regeneration: A New Future for Dental and Biomedical Applications

**DOI:** 10.3390/ma16186238

**Published:** 2023-09-15

**Authors:** Nina Attik, Rémy Gauthier, Mohammed Ahmed

**Affiliations:** 1 Faculté d’Odontologie, Université Claude Bernard Lyon 1, Université de Lyon, 69372 Lyon, France; 2 CNRS, INSA de Lyon, UCBL, MATEIS UMR CNRS 5510, Lyon, Bât. Saint Exupéry, 23 Av. Jean Capelle, 69621 Villeurbanne, France; 3 Department of Dental Biomaterials, Tanta University, Tanta 31527, Egypt

In order to enhance and promote tissue repair and healing processes, current exploratory and investigative research lines in medical and dental treatments are focusing on the use of bioactive materials that are able to induce and trigger a specific targeted biological activity to stimulate the suitable response from the host tissue.

In this context, bioactive glasses (BAGs) could be classified as “bioactive materials” because of their ability to interact with biological tissues through formation of a hydroxyapatite-rich layer at the BAG–tissue interfaces, and encourage tissue remineralization, regeneration and integration. One of the potential interactions is the formation of a chemical bond with bone, or at least, promoting its self-healing potential through cell stimulation. Since the introduction of BAGs in 1969 by Professor Larry Hench, the development of BAG-based materials has continued to rise for diverse clinical indications for minimally invasive and biologically based therapies [1] such as:

- In oral hygiene: Novamin^®^ (NovaMin Technology, Alachua, FL, USA) and BiominF^®^ (Biomin Holding GmbH, Pottenbrunn, Austria) are examples of two developed toothpastes containing BAGs (with or without Fluor) for enamel remineralisation and dental hypersensitivity/tooth sensitivity [2].
- In endodontics: BAG-based pulp capping materials could stimulate pulp cells’ secretory potential, increase the sealing ability, decrease bacterial infiltration, and preserve pulp vitality.
- In restorative dentistry: BAGs can be potentially added to dental adhesives and composites. BAG-based restorative materials are expected to possess remineralizing potential or at least to provide a certain degree of anti-enzymatic/bacterial activity with sustained durability over time.
- In bone healing: Some products containing BAG particles are already available in the market such as bone filling materials (Biogaran^®^ (Biogaran, Colombes, France)-Perioglas^®^ (Novabone Products LLC, Alachua, FL, USA)) or implant coatings to improve osteointegration. Current research lines are focused on the development of BAG-based bone scaffolds to promote bone regeneration through in vivo tissue engineering [3].

Although the majority of BAG-based dental materials are still in the experimental phase, this Special Issue aims to explore and elucidate the current status and future perspectives related to BAG use in dental biomaterials and highlight the rationale and benefits behind their applications in the medical as well as dental fields.
